# A “Fundamentals” Train-the-Trainer Approach to Building Pediatric Critical Care Expertise in the Developing World

**DOI:** 10.3389/fped.2018.00095

**Published:** 2018-04-27

**Authors:** Sheri S. Crow, Beth A. Ballinger, Mariela Rivera, David Tsibadze, Nino Gakhokidze, Nino Zavrashvili, Matthew J. Ritter, Grace M. Arteaga

**Affiliations:** ^1^Pediatric Critical Care, Mayo Clinic, Rochester, NY, United States; ^2^Department of Surgery, Division of Trauma, Acute Care General Surgery and Surgical Critical Care, Mayo Clinic, Rochester, NY, United States; ^3^Head of Maternal and Child Health Department, EVEX Medical Corporation, Tbilisi, Georgia; ^4^David Tvildiani Medical University, Tbilisi, Georgia; ^5^Department of Anesthesia and Critical Care Medicine, Mayo Clinic, Rochester, NY, United States

**Keywords:** education, train-the-trainer, pediatric critical care, training, pediatric fundamental critical care support

## Abstract

Pediatric Fundamental Critical Care Support (PFCCS) is an educational tool for training non-intensivists, nurses, and critical care practitioners in diverse health-care settings to deal with the acute deterioration of pediatric patients. Our objective was to evaluate the PFCCS course as a tool for developing a uniform, reproducible, and sustainable model for educating local health-care workers in the optimal management of critically ill children in the Republic of Georgia. Over a period of 18 months and four visits to the country, we worked with Georgian pediatric critical care leadership to complete the following tasks: (1) survey health-care needs within the Republic of Georgia, (2) present representative PFCCS lectures and simulation scenarios to evaluate interest and obtain “buy-in” from key stakeholders throughout the Georgian educational infrastructure, and (3) identify PFCCS instructor candidates. Georgian PFCCS instructor training included the following steps: (1) US PFCCS consultant and content experts presented PFCCS course to Georgian instructor candidates. (2) Simulation learning principles were taught and basic equipment was acquired. (3) Instructor candidates presented PFCCS to Georgian learners, mentored by PFCCS course consultants. Objective evaluation and debriefing with instructor candidates concluded each visit. Between training visits Georgian instructors translated PFCCS slides to the Georgian language. Six candidates were identified and completed PFCCS instructor training. These Georgian instructors independently presented the PFCCS course to 15 Georgian medical students. Student test scores improved significantly from pretest results (*n* = 14) (pretest: 38.7 ± 7 vs. posttest 62.7 ± 6, *p* < 0.05). A Likert-type scale of 1 to 5 (1 = not useful or effective, 5 = extremely useful or effective) was used to evaluate each student’s perception regarding (1) relevance of course content to clinical work students rated as median (IQR): (a) relevance of PFCCS content to clinical work, 5 (4–5); (b) effectiveness of lecture delivery, 4 (3–4); and (c) value of skill stations for clinical practice, 5 (4–5). Additionally, the mean (±SD) responses were 4.6 (±0.5), 3.7 (±0.6), and 4.5 (±0.6), respectively. Training local PFCCS instructors within an international environment is an effective method for establishing a uniform, reproducible, and sustainable approach to educating health-care providers in the fundamentals of pediatric critical care. Future collaborations will evaluate the clinical impact of PFCCS throughout the Georgian health-care system.

## Introduction

The failure to recognize and appropriately intervene in the early presentation of critical illness results in significant morbidity and mortality worldwide (1, 2). Patient outcomes could be dramatically improved through the application of basic resuscitation and stabilization principles (3, 4). Initiatives seeking to successfully spread this high impact medical knowledge to resource-limited health-care settings face significant challenges that are difficult to overcome during short-term visits (5). Overcoming language barriers, relationship building with key local stakeholders, and understanding the intricacies of the cultural environment are critical pre-requisites for implementing and sustaining any educational initiative (6). Recognizing that local providers and leaders are best suited to overcome these challenges (7), we sought an effective tool for teaching acute care management principles to both community and critical care providers within the Republic of Georgia.

Health-care leaders in the Republic of Georgia and the Ministry of Health approached physicians at our institution to explore opportunities for improving Georgian maternal and child health outcomes through education. The Republic of Georgia, through a colleague who completed her professional training at our institution, sought assistance in developing a uniform sustainable approach to educating health-care workers in the acute care management of children throughout the Republic of Georgia.

In 1991, the country’s independence from the Soviet Union provided an unprecedented opportunity to transform Georgian health care from the Soviet model (Semashko model) to a new health-care system. The original transition period trialed both free health care to all citizens and privatized medicine, before establishing the current system in 2013 of government funded health care that is distributed through a state agency. Under this model the state finances health care, but the delivery is largely reliant on private medical facilities and personnel. Simultaneous changes in the Georgian political climate, which now permitted unprecedented access to Western medicine, generated growing interest in restructuring the country’s health-care education to align with current evidence based practices (8, 9).

**Figure 1:**
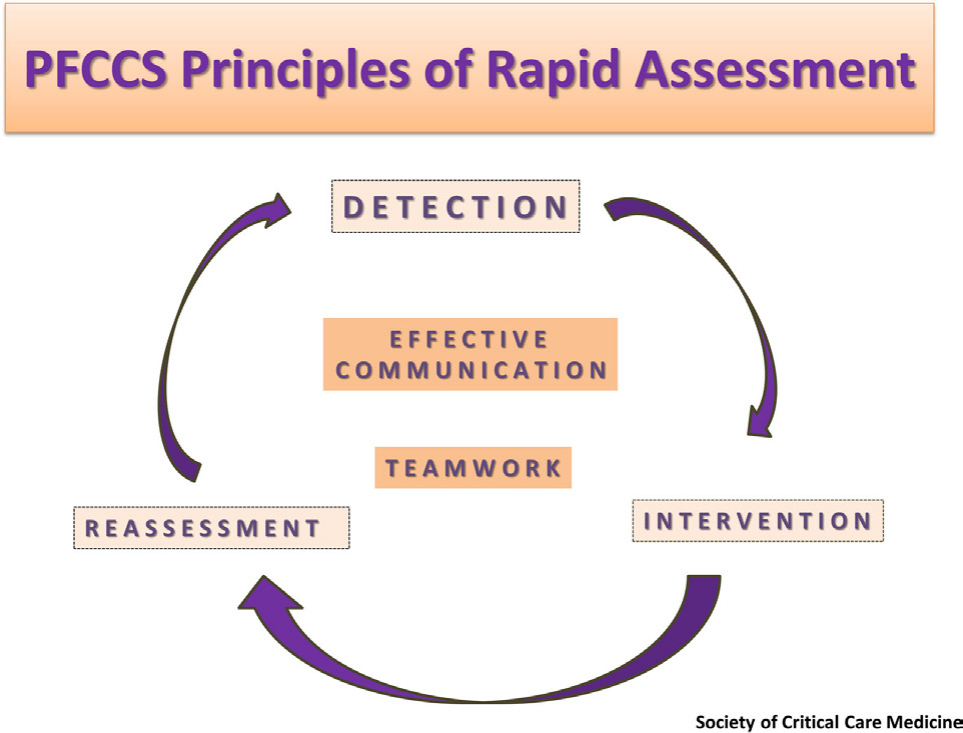
Timeline for instructor training with specific goals for each visit.

The Society of Critical Care Medicine (SCCM) has developed and standardized adult and pediatric courses that teach the fundamentals of critical care management through didactic lectures and hands-on simulation scenarios. The Pediatric Fundamental Critical Care Support (PFCCS) is intended to train non-intensivists, nurses, and critical care providers. The main objective is to provide an educational framework to address recognition and initial management of the clinically unstable pediatric patient and to accurately identify the need for expert consultation and/or transfer to a higher level of care (Figure 1) (10). These goals make the PFCCS course an ideal educational tool for advancing critical care expertise in resource-rich and limited areas where training in critical care management is needed (11–13). The course management principles are applicable to all patients, regardless of the resources available within the health-care setting to which they present.

In collaboration with Georgian health-care leaders, a group of multidisciplinary intensivists from the Mayo Clinic utilized a “train the trainer” approach to establish a PFCCS course through a series of short-term visits to the Republic of Georgia (14, 15). The main objective was to train Georgian health-care providers as PFCCS instructors to promote the development of a consistent, uniform, and sustainable approach to the management of critically ill children throughout the country.

## Materials and Methods

Over 18 months and four visits, Mayo Clinic and the Georgian health-care providers collaborated to achieve the following goals: (1) survey the health-care needs within the Republic of Georgia, (2) preview PFCCS lectures and simulation scenarios throughout the Georgian health-care infrastructure to obtain “buy-in” from key stakeholders, and (3) train Georgian health-care workers as PFCCS instructors. Leadership organizations supporting the Georgian PFCCS Development included the Georgian Ministry of Health, United Nations Children’s Fund (UNICEF), United Nations Population Fund (UNFPA), United States Agency for International Development (USAID), Centers for Disease Control and Prevention (CDC), and Mayo Clinic Global Health.

The initial needs assessment visit to Georgia included tours of tertiary hospitals providing pediatric critical care and maternal birthing centers. Tours included on-site assessment of equipment resources along with demonstrations of local resuscitation protocols. At each location, Mayo team members presented samples of PFCCS course material to determine interest and relevance of the material to the Georgian health-care system. Meetings were held with the Minister of Health and representatives from UNICEF, UNFPA, and USAID to understand the maternal and child health challenges and the existing efforts to address them.

At the conclusion of the needs assessment visit, through discussions with Georgian health-care leadership the following objective was established: Train local health-care providers as PFCCS instructors to promote the development of a uniform, reproducible, and sustainable approach to the management of acutely ill children throughout Georgia. Once trained, Georgian PFCCS instructors would educate clinicians at every point of care within the health-care system (regional hospitals and clinics, transport teams, and tertiary centers). This approach would have the added benefit of strengthening relationships between health-care providers in tertiary and regional settings thereby enhancing access to critical care expertise across the country.

### Instructor Selection and Training

To optimize the success of Georgian PFCCS course development, the initial instructor candidates were selected based on specific criteria. Candidates chosen were established, influential providers within local health-care communities with experience providing critical care. There were no training programs specific to critical care in Georgia, so instructor candidates were physicians practicing in the disciplines of pediatric, neonatal, and emergency medicine. English fluency was required to assure optimal didactic understanding and translation of the PFCCS course materials (textbooks and slide sets) which we could only offer in English.

**Figure 2:**
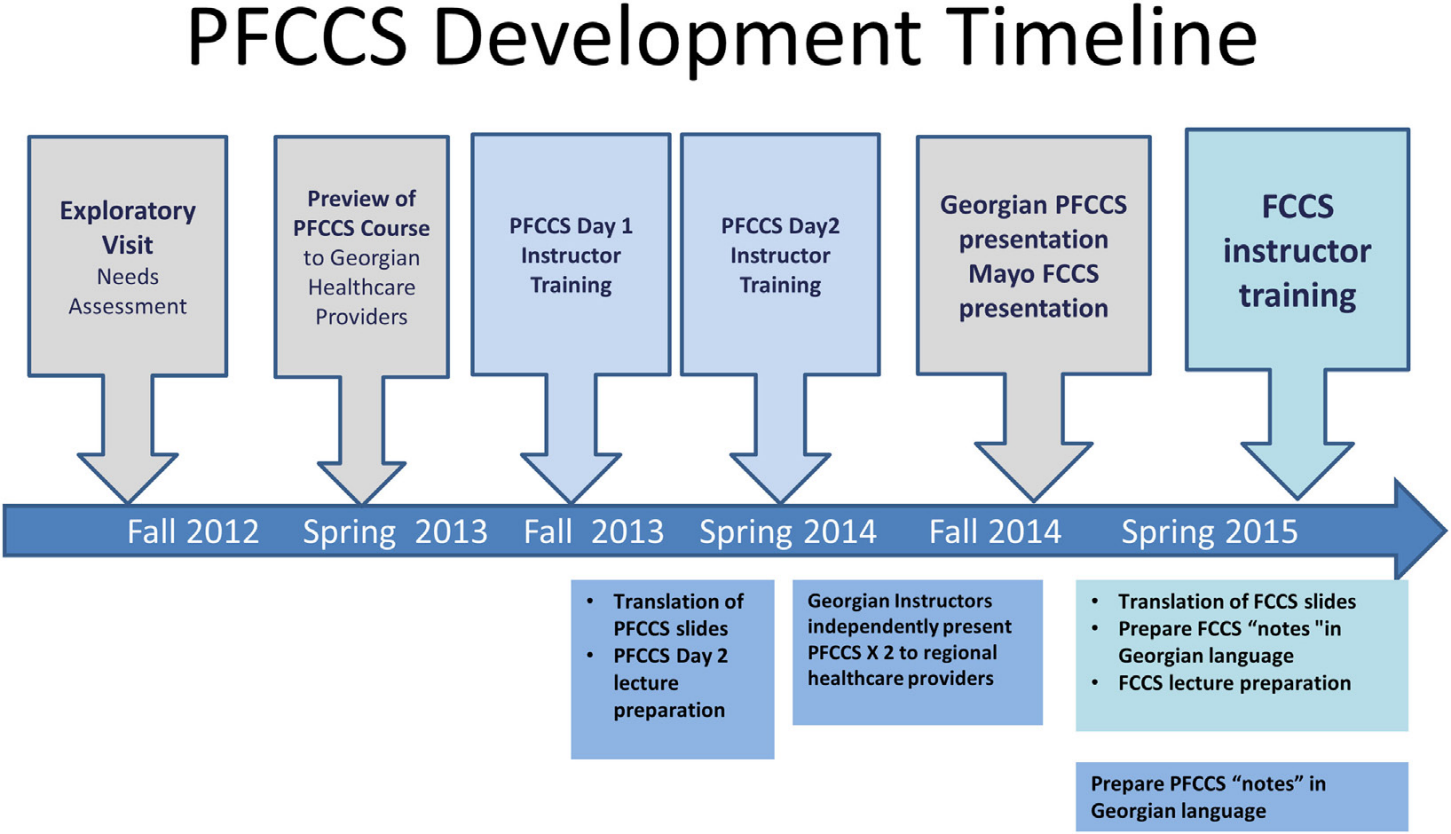
The PFCCS principles of rapid assessment emphasized during the course.

Training of PFCCS instructor candidates is outlined in Figure 2 and was accomplished through the following steps consistent with SCCM guidelines for achieving instructor certification (10): (1) US PFCCS consultants presented the PFCCS course in its entirety to the Georgian instructor candidates, (2) US PFCCS consultants and Georgian instructor candidates spent 1 day focused on adult learning principles, practicing hands-on simulated case scenarios, and rehearsing lectures, (3) instructor candidates presented the PFCCS course to Georgian students while mentored by US PFCCS consultants. (4) US consultants provided individual feedback to each instructor candidate based on PFCCS course evaluation forms completed by the students and US PFCCS consultants. (5) Georgian candidates translated the PFCCS slides for their lectures into the Georgian language so that future course presentations could be provided to learners in their native tongue.

At the conclusion of Georgian instructor training, a course director and administrator were identified from the candidate pool. The job description and responsibilities for these positions were outlined and the next steps and expectations were established in collaboration with the Georgian and US leadership. The process for obtaining a Georgian PFCCS course license was reviewed with the newly identified course administrator and director. Prior to this, all training related PFCCS presentations and materials were provided free of charge through the multi-course license purchased by the US consultants home institution. Finally, the appropriate paperwork was completed and submitted to SCCM to obtain official instructor certificates for each Georgian instructor candidate meeting all usual SCCM requirements (10).

### Developing Sustainability and Transitioning to Independence

In order to build confidence, optimize instructor retention, and promote dissemination of the course throughout Georgia, US instructors returned to Georgia at 6 month intervals. With each visit, US team members proctored Georgian led PFCCS courses, and dedicated a day during each visit to discuss successes and challenges encountered by Georgian instructors between visits. Additional skill training in simulated learning and educational methods, such as debriefing, were taught and practiced together. Finally, administrative processes were reviewed and goals established for ongoing course maintenance and expansion throughout Georgia.

Nurturing independence and maintaining momentum and enthusiasm for the Georgian PFCCS course between the US provider visits was critical to establishing sustainable infrastructure that would function independently of US collaborators. The PFCCS course case-based, interactive, and hands-on approach were novel concepts to the Georgian medical education culture. Therefore, it was important for the Georgian instructors to practice these new presentation and teaching skills. Toward the goal of fostering independence, Georgian instructors were expected to teach a minimum of two courses between US visits, and videotape one presentation to permit remote viewing and feedback.

### Regional Dissemination

With the first generation of Georgian instructors established, the collaboration turned to expanding the instructor pool within the country. Regional health-care workers became the next target in order to exponentially increase opportunities for course dissemination and foster adoption of the fundamental principles of patient management across the country. In this phase, at each US visit, Georgian and US instructors presented samples of the PFCCS course within each regional health-care setting. Following each presentation, Georgian leadership identified and invited health-care providers within those regions to pursue instructor training and provided travel and accommodations for candidates to convene at a centralized training site. The Georgian PFCCS instructors then independently trained these new candidates by repeating the steps used in their own training process, under the mentorship of the US consultants. US consultants were paired with an interpreter to observe every aspect of the training process. Specifically, they evaluated the integrity of the course material presented and provided feedback and encouragement to the Georgian instructors as they trained in the new candidates. The interpreters facilitating this process were Georgian physicians educated within both Georgian and United States training programs. The same interpreters were employed for every US visit to assure continuity in the assessment of program development and accuracy in translation of the course material and concepts.

### Statistical Analysis

For the test scores in both the pretest and in the posttest data, a mean with SD was calculated. The Likert-type scale included nominal data collected and was evaluated using a mean ± SD statistical. We chose median (IQR) as the set of survey data analysis to describe the value because of the small sample size of the learners. To highlight the clarity, mean (±SD) values are added as well.

## Results

Seven Georgian health-care providers were identified and completed the first PFCCS instructor training course in Georgia. Six candidates became SCCM certified PFCCS instructors and the seventh participant was selected as the course administrator. These instructor candidates presented the PFCCS course to 15 Georgian medical students with no prior training in critical care and limited clinical experience. The success of the Georgian instructors in teaching the PFCCS principles of management was demonstrated by significant improvement in the learner’s test scores (*n* = 14) (pretest: 38.7 ± 7 vs. posttest 62.7 ± 6, *p* < 0.05).

This first class of PFCCS students was surveyed following the course presentation. Responses were based on a Likert-type scale of 1–5 (1 = not useful; 5 = extremely useful). These 14 students rated as median (IQR): (a) relevance of PFCCS content to clinical work, 5 (4–5); (b) effectiveness of lecture delivery, 4 (3–4); and (c) value of skill stations for clinical practice, 5 (4–5). Additionally, the mean (±SD) responses were 4.6 (±0.5), 3.7 (±0.6), and 4.5 (±0.6), respectively, denoting that relevance of content and value of skills stations were highly rated.

At the completion of their training, the Georgian instructors reported enhanced communication and teamwork surrounding resuscitation events and improved consistency in the application of evidence based principles to clinical management. Furthermore, the instructors believed the course had positively impacted their approach to education within their health-care communities. Specifically, the PFCCS format had improved their presentation skills, ability to increase audience engagement, and introduced them to simulation-based learning methods that were now being utilized for teaching within each instructor’s local health-care setting. Finally, instructors reported that PFCCS textbooks were a highly utilized and sought after clinical reference book.

As PFCCS training continued, Georgian instructors began seeking opportunities to establish measurable criteria for hospital performance that would objectively demonstrate the benefit of PFCCS training on patient outcomes. These requests highlighted the need to parallel the PFCCS medical education with an introduction to quality improvement (QI) methods. Thereafter, visits included instruction in QI methodology and identifying relevant applications for QI models such as the Plan-Do-Study-Act cycles. Together, we explored goals and walked through a plan (P) to test the initial goal, introduce the change (D), observe and collect results (S), and identify modifications necessary to achieve the initial goal (A). Specific projects discussed focused on infection prevention, interdisciplinary communication, and protocol compliance.

**Figure 3:**
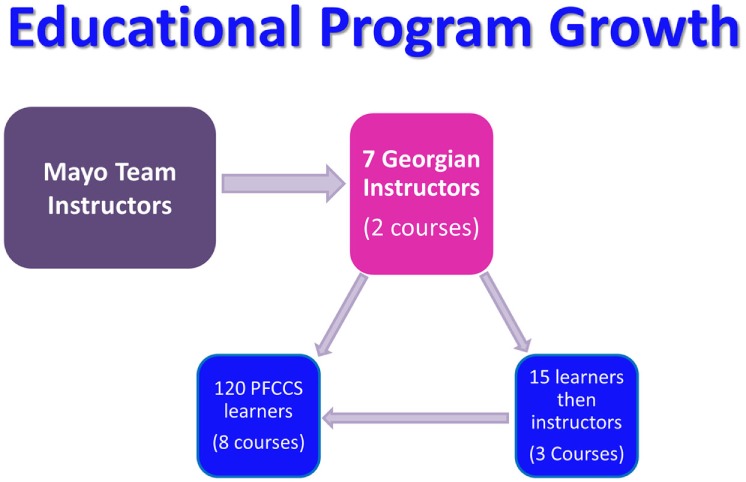
Program growth within local resources at the completion of six visits.

The original six Georgian PFCCS instructors have now trained an additional nine instructors. These 15 individuals, representing diverse regions and health-care systems throughout Georgia, have independently presented PFCCS 8 times to more than 120 health-care workers (Figure 3). Establishing a Georgian PFCCS instructor network has positively impacted other educational programs within the Georgian health-care system. Local efforts to develop Pediatric Advance Life Support courses within Georgia were already underway when PFCCS was first introduced.

## Discussion

There are many advantages to leveraging an educational platform such as PFCCS to support international collaborations. In our experience, PFCCS promoted rapid relationship building between US and Georgian health-care providers. The SCCM sponsorship and oversight of the course provides information deemed important by an internationally recognized organization rather than any one individual or specific institution. The interactive lecture format and case scenarios provide a non-threatening framework for rapidly understanding existing practice patterns, resource limitations, and potential barriers to change within a foreign health-care environment. PFCCS material is presented to learners as being relevant to all health-care settings (resource abundant and resource limited) and therefore unifies international and local collaborators around a common cause.

PFCCS teaches principles that may not only be considered best practices within US systems, but may also represent novel ideas and concepts within certain health-care environments. The course framework facilitates non-threatening discussion of these ideas while respectfully highlighting the cultural diversity between the instructor and student’s health-care environments. Case discussions unite instructor and student around universal clinical challenges, thereby accelerating relationship building and improving receptiveness to the course objectives. For example, PFCCS development in Georgia introduced interosseous (I/O) needles as a method for gaining rapid intravenous access during resuscitation. The suggestion of an I/O needle during our initial PFCCS presentations was met with shock and dismissed by the Georgian health-care providers in attendance. On future trips, we brought the intraosseous equipment to demonstrate the ease of use and the lifesaving potential of this equipment. The PFCCS instructors were the first Georgians to adopt the idea. Their acceptance of the I/O as a valuable medical tool was the gradual result of continuing to teach about I/O application within the context of the PFCCS case scenarios. Ultimately, one of the PFCCS instructors, the director of Pediatric Emergency Medicine training in Georgia, has decided to implement I/O needles as part of her PhD thesis. The research for her thesis involves the pilot introduction of I/O use in the emergency department and pediatric intensive care unit of a major children’s hospital in Georgia.

The PFCCS course approach to addressing common pediatric emergencies creates a platform to promote interdisciplinary communication and team dynamics (Figure 1). The simulation-based medical scenarios provide an excellent venue for promoting and practicing positive culture changes to improve collective decision making and ultimately patient care. Health-care delivery in these hands-on sessions is presented as an interdisciplinary team collaboration rather than an individual performance, highlighting the critical role played by both physician and non-physician team members. In Georgia, gradual cultural shifts stemming from these PFCCS teamwork principles, have translated to inviting nurses to join physicians in becoming PFCCS instructor candidates. Inclusion of nursing leadership in the PFCCS instructor network has accelerated adoption of PFCCS principles at the bedside, improved interdisciplinary communication, and led to lifesaving changes in health-care delivery throughout the Georgian health-care system.

Fundamental concepts for enhancing team dynamics include mutual respect and creating a shared mental model. These concepts are explicitly taught and promoted in the debriefing sessions that follow PFCCS simulation scenarios. These discussions play an essential role in establishing a psychologically safe framework where learners can reflect on their actions, identify performance gaps, and discuss areas for personal and process improvements. The opportunity for transparent discussion of patient management was unprecedented in the Georgian medical culture. Following the first introduction to debriefing, Georgian PFCCS instructor candidates expressed immense gratitude for the opportunity to safely ask questions regarding medical decisions the team made during the case scenario, while receiving constructive feedback specific to their management. Eclipsing all this was the opportunity to recognize they were not alone in their feelings of uncertainty or the emotional turmoil that surrounds the care of a critically ill child. The impact of these sessions on our Georgian colleagues impressed upon us the importance of continuing to pursue debriefing opportunities within our own US health-care environment as an effective method for supporting team members and enhancing team dynamics.

Although PFCCS proved to be effective in achieving our educational objectives there are several inherent limitations to this approach that must be addressed. First, it must be acknowledged that PFCCS is one of many courses to utilize the “train the trainer” approach. The American Red Cross and American Heart Association, to name a few, have a long history of educating the world in optimal resuscitation techniques. These forerunners lent support to our idea that this educational model would also be effective for enhancing critical care knowledge in diverse settings. Unfortunately, the most important barrier to developing any of these standardized courses in a developing country is that the cost for training materials and licensing are often prohibitive. These financial barriers limit the feasibility of sustaining the courses over the long run in economically challenged settings. Finally, although trainees found the course useful, we have yet to establish that PFCCS training of health-care providers increases the survival of critically ill children in Georgia. However, the training process led to the country’s adoption of QI methodology and implementation of the interosseous needle. The potential for these changes to positively impact patient outcome are well documented in the medical literature.

In addition to the course limitations listed above, we encountered a number of logistic challenges that had to be overcome to successfully achieve our collaborative goals. The language barrier was perhaps the most difficult challenge encountered in the training process. At the time of our first visit, we encountered limited English fluency throughout Georgia. The PFCCS textbook and slides are written in English and time and cost made initial translation of the materials to Georgian infeasible. Instead, we selected PFCCS instructor candidates fluent in English. After gaining permission from the SCCM PFCCS leadership, each instructor translated their lecture into the Georgian language. This step was an essential work around as English fluency was even less common in the regional areas that have the greatest need for critical care education. Unfortunately, although the course slides have now been translated to Georgian and the course is taught in Georgian, the textbooks are still written in English, thereby limiting this reference as a tool in clinical management for a large percentage of the Georgian health workforce. Efforts are underway to translate the textbook into Georgian. In the interim, English fluency within the Georgian population continues to increase.

The PFCCS course utilizes a combination of case-based lectures and hands-on simulated sessions to reinforce course objectives. The first Georgian instructor candidates had limited experience with simulated case-based learning prior to their PFCCS training. To develop this skill set, extended simulation practice sessions were part of every US visit, and included real-time debrief and training in simulation teaching methods and principles of adult learning. Between visits, instructors were required to lead a minimum of two simulated teaching sessions within their individual place of work. These “practice sessions” raised instructor confidence in simulated teaching and inadvertently changed their approach to training the local medical teams they supervised.

Financing educational programs within low-income countries is always a challenge and often places significant limitations on successful achievement of project goals. Training PFCCS instructors in a foreign health-care system is labor intensive for both the local work force and the international collaborators. The perceived financial cost to developing internationally recognized courses such as PFCCS is often seen as prohibitive for resource-limited locations. PFCCS is not a free educational resource. A license purchased through SCCM is required to access and present the course lectures and training materials. The license cost for a country is determined by a tiered structure and is therefore available at a markedly reduced rate for resource-limited countries (10). However, successful international collaborations that lead to sustainable changes in health-care environments are rare. Recognizing the uncertainty in the feasibility of achieving our objective we sought to minimize the financial cost of this project to our hosts. We asked our Georgian stakeholders and instructor candidates to invest their time and effort in course development without financial risk or obligation. Institutions can purchase a multi-course licenses through SCCM to permit unlimited course presentations in any location they choose. The US PFCCS team under the Mayo Clinic institutional license was able to present the PFCCS course to Georgian instructor candidates free of charge. US instructors covered their own travel costs and donated PFCCS textbooks to reduce the financial cost for development even further. Once the Georgian instructors were certified by SCCM and independently teaching PFCCS in Georgia, they purchased their own multi-course license. At this stage of development, the success of the initiative and value of the program to Georgian health care was well recognized by the Georgian Ministry of Health and the non-government organizational sectors. As a result, Georgian PFCCS leadership was able to secure financial support from local sources and UNICEF, CDC, and UNFPA. Regular meetings with these entities during each US visit to Georgia was critical to maintaining enthusiasm for the project and ultimately financial support. Furthermore, as recognition of the course value grew, opportunities for offering PFCCS to surrounding countries is creating new opportunities for generating revenue to sustain the course and promote the international visibility of the Georgian health-care developments.

Perhaps, the most significant outcome of PFCCS development in Georgia were the relationships developed between US and Georgian health-care teams. Successful certification of Georgian PFCCS instructors increased enthusiasm for considering additional educational courses such as the adult Fundamental of Critical Care Support (FCCS) course and Advanced Trauma and Life Support. Recognizing that the majority of providers in Georgia are treating primarily adults, the efforts to establish FCCS in Georgia proceeded rapidly. The number of clinicians seeking to become FCCS instructors more than doubled that of the original seven PFCCS instructor candidates. FCCS candidates were required to complete the PFCCS course, taught by their pediatric colleagues, who were now certified PFCCS instructors. The PFCCS instructors approached this process with great trepidation. These instructors disclosed to the US consultants that the FCCS instructor candidates were actually their former teachers, each a highly respected anesthesiologist or internist within the Georgian health-care community. Despite their initial concerns, the PFCCS instructors delivered the course seamlessly. At the conclusion, FCCS candidates were overcome with pride in their former students and marveled at the effectiveness of the course format for teaching critical care management and developing instructors into highly competent teachers. To date, there are more than 50 FCCS instructors in Georgia representing every medical discipline and region. The PFCCS and FCCS courses are the first Georgian educational programs to qualify for continuing medical education (CME) credit. The Georgian FCCS director, Dr. Marika Toidze, plans to train an additional 80 FCCS instructors in 2018 to accommodate the new CME requirements in Georgia.

In summary, our experience confirms those reported elsewhere that high impact, sustainable changes in patient management can be accomplished through education that occurs independent of expensive investments in material resources (12, 13). An established and internationally recognized educational program such as PFCCS is a successful method for developing international collaborations with long term benefit through a series of short-term visits. The PFCCS interactive format permits efficient exchange of information to pursue focused needs assessment and understanding of the local infrastructure strengths and challenges. The learning objectives are relevant to all health-care settings (resource abundant and resource limited) and therefore unify international and local collaborators around a common cause. The original request of the US consultants was to help improve maternal and child health-care delivery. We have found PFCCS to be an effective tool for taking the first steps toward that objective. Furthermore, it has helped create a foundational vocabulary amongst stakeholders as we move forward to address other aspects of health-care infrastructure, including medical transport and trauma system development that impact health outcomes for the Georgian population.

## Conclusion

The SCCM Fundamental’s “train the trainer” approach provided a uniform, reproducible, and sustainable framework for educating health-care providers in the fundamentals of pediatric critical care throughout the Republic of Georgia. Developing local PFCCS instructors within an international health-care environment is an effective model for establishing durable educational infrastructure that functions independently of international involvement.

## Ethics Statement

This manuscript describes and educational initiative in another country. Although data are presented regarding the project outcomes, this was not an intervention study or a research project and therefore did not require IRB review. Please let us know if the paper should be submitted under a different format than as an “original research article.”

## Author Contributions

All authors contributed to the conception and design of the study and participated in the training processes described in this manuscript; BB and GA organized the database; GA performed the statistical analysis; SC wrote the first draft of the manuscript with extensive contribution from BB; GA wrote sections of the manuscript. All authors contributed to manuscript revision, read and approved the submitted version.

## Conflict of Interest Statement

The authors declare that this work was carried out without any personal, professional, or financial relationships that could potentially be construed as conflicts of interest.
